# GGC expansions in *NOTCH2NLC* contribute to Parkinson disease and dopaminergic neuron degeneration

**DOI:** 10.1111/ene.16145

**Published:** 2023-11-17

**Authors:** Qiong Liu, Juan Chen, Jin Xue, Xun Zhou, Yun Tian, Qiao Xiao, Wen Huang, Yongcheng Pan, Xiaoxia Zhou, Jian Li, Yuwen Zhao, Hongxu Pan, Yige Wang, Runcheng He, Yaqin Xiang, Tian Tu, Qian Xu, Qiying Sun, Jieqiong Tan, Xinxiang Yan, Jinchen Li, Jifeng Guo, Lu Shen, Ranhui Duan, Beisha Tang, Zhenhua Liu

**Affiliations:** ^1^ Department of Neurology, Xiangya Hospital Central South University Changsha China; ^2^ Key Laboratory of Hunan Province in Neurodegenerative Disorders Central South University Changsha China; ^3^ Center for Medical Genetics and Hunan Key Laboratory of Medical Genetics, School of Life Sciences Central South University Changsha China; ^4^ Department of Geriatrics, Xiangya Hospital Central South University Changsha China; ^5^ Department of Nuclear Medicine, Xiangya Hospital Central South University Changsha China; ^6^ National Clinical Research Center for Geriatric Disorders, Xiangya Hospital Central South University Changsha China

**Keywords:** dopaminergic neuron degeneration, GGC repeat expansions, *NOTCH2NLC*, Parkinson disease

## Abstract

**Background and purpose:**

The role of GGC repeat expansions within *NOTCH2NLC* in Parkinson's disease (PD) and the substantia nigra (SN) dopaminergic neuron remains unclear. Here, we profile the *NOTCH2NLC* GGC repeat expansions in a large cohort of patients with PD. We also investigate the role of GGC repeat expansions within *NOTCH2NLC* in the dopaminergic neurodegeneration of SN.

**Methods:**

A total of 2,522 patients diagnosed with PD and 1,085 health controls were analyzed for the repeat expansions of *NOTCH2NLC* by repeat‐primed PCR and GC‐rich PCR assay. Furthermore, the effects of GGC repeat expansions in *NOTCH2NLC* on dopaminergic neurons were investigated by using recombinant adeno‐associated virus (AAV)‐mediated overexpression of *NOTCH2NLC* with 98 GGC repeats in the SN of mice by stereotactic injection.

**Results:**

Four PD pedigrees (4/333, 1.2%) and three sporadic PD patients (3/2189, 0.14%) were identified with pathogenic GGC repeat expansions (larger than 60 GGC repeats) in the *NOTCH2NLC* gene, while eight PD patients and one healthy control were identified with intermediate GGC repeat expansions ranging from 41 to 60 repeats. No significant difference was observed in the distribution of intermediate *NOTCH2NLC* GGC repeat expansions between PD cases and controls (Fisher's exact test *p*‐value = 0.29). Skin biopsy showed P62‐positive intranuclear NOTCH2NLC‐polyGlycine (polyG) inclusions in the skin nerve fibers of patient. Expanded GGC repeats in *NOTCH2NLC* produced widespread intranuclear and perinuclear polyG inclusions, which led to a severe loss of dopaminergic neurons in the SN. Consistently, polyG inclusions were presented in the SN of EIIa‐NOTCH2NLC‐(GGC)98 transgenic mice and also led to dopaminergic neuron loss in the SN.

**Conclusions:**

Overall, our findings provide strong evidence that GGC repeat expansions within *NOTCH2NLC* contribute to the pathogenesis of PD and cause degeneration of nigral dopaminergic neurons.

## INTRODUCTION

As the second most frequent neurodegenerative disorder, Parkinson disease (PD) is characterized by motor symptoms, such as bradykinesia, resting tremor, muscle stiffness, and postural instability, as well as nonmotor symptoms like olfactory dysfunction, sleep disorders, constipation, and dysautonomia. These symptoms are caused by the loss of neurons in various brain regions and may occur before or after the loss of dopaminergic neurons, which are commonly affected in PD [1, 2]. PD is thought to be caused by a combination of ageing and genetic and environmental risk factors. To date, more than 20 genes with different degrees of genetic evidence have been found to be mutated in monogenic PD [3, 4, 5, 6]. Although progress has been made, much remains unclear about the genetic etiology of the PD.

In recent years, there has been growing interest in diseases caused by short tandem repeat expansions, which have been associated with various neurodegenerative disorders [7, 8]. GGC repeat expansions in the *GIPC1* locus have been screened as candidate gene for idiopathic PD, as they have been found to be associated with other movement disorders and essential tremor [9, 10, 11]. Previously, our group and others identified GGC repeat expansion in the *NOTCH2NLC* gene as the genetic cause of neuronal intranuclear inclusion disease (NIID) [12, 13, 14]. Subsequently, *NOTCH2NLC* with GGC repeat expansions has also been identified in other neurodegenerative disorders [15, 16, 17]. We also reported three different parkinsonism families (3/205) with *NOTCH2NLC* GGC repeat expansion, indicating that the GGC repeat expansions in *NOTCH2NLC* could contribute to the pathogenesis of PD [12], which was verified by other group [18]. However, the pathogenic GCC repeat expansions within *NOTCH2NLC* were rare and were not associated with PD in the European population [19, 20, 21]. Further studies with larger case–control cohorts will help to define the role of GGC repeat expansions within *NOTCH2NLC* in PD. In this study, one of the main goals is to establish the prevalence of the *NOTCH2NLC* GGC repeat expansions in a large number of case–control individuals to further assess whether these repeat expansions are associated with PD.

With the progress in genetic identification that has been made, molecular pathogenesis of *NOTCH2NLC* GGC repeat expansions is attracting considerable attention [22, 23, 24, 25]. However, the pathology of PD patients with the *NOTCH2NLC* GGC repeat expansions is still poorly understood, and the role of *NOTCH2NLC* with expanded GGC repeats in substantia nigra (SN) dopaminergic neurons remains unclear [12, 17]. Here, we examined the effects of GGC repeat expansions within *NOTCH2NLC* on dopaminergic neurons by expressing *NOTCH2NLC* with 98 GGC repeats via recombinant adeno‐associated viruses (AAVs) in the SN of mice.

## METHODS

### Standard protocol approvals, registrations, and patient consents

The study was approved by the ethics committee of Xiangya Hospital Central South University. All participants provided written informed consent.

### Participants and clinical assessment

Participants were recruited between October 2006 and January 2019 at the Xiangya Hospital Central South University and at other cooperating hospitals of the Parkinson's Disease and Movement Disorders Multicenter Database and Collaborative Network in China (PD‐MDCNC; http://pd‐mdcnc.com). All participants fulfilled the Movement Disorders Society clinical diagnostic criteria for PD [26].

Genomic DNA of peripheral blood leukocytes was obtained from these PD patients, their available family members, and 1085 unaffected healthy control subjects. A comprehensive dataset of basic demographic data, including the subjects' age, gender, family history, disease duration, and clinical features including motor and nonmotor manifestations, was collected from the PD patients enrolled in this study and inputted into the PD‐MDCNC.

### Polymerase chain reaction assays and GGC repeat size determination

Repeat‐primed polymerase chain reaction (PCR) and GC‐rich PCR (GC‐PCR) were conducted for detecting CGG repeat expansions in *NOTCH2NLC*. Primers were used as previously reported [12, 15]. A fluorescein (FAM)‐labeled gene‐specific primer (5′‐CCT CAG CCC GAT ACT CAC CAT‐3′) and repeat‐containing primers (5′‐TAC CAA TAC GCA TCC CGC GAT TTG TCT TA [CGG]‐5–3′) were utilized for identifying the CGG repeat expansion. For the GC‐PCR assay, the FAM‐labeled forward primer (5′‐AGC GCC AGG GCC TGA GCC TTT GAA GCA G‐3′) and reverse primer (5′‐TCG CCC CAG GTG GCA GCC CCG GGC GCC GCG GAC‐3′) were utilized for repeat size determination.

### Animals

The EIIa‐NOTCH2NLC‐(GGC)_17_ and EIIa‐NOTCH2NLC‐(GGC)_98_ transgenic mice, in which *NOTCH2NC* with normal GGC repeats (17 GGC) or expanded GGC repeats (98 GGC) was expressed ubiquitously, were established in our previous study [22]. The transgenic mice and wild‐type C57BL/6J mice were bred and maintained on a 12:12‐h light/dark cycle (lights off at 7 p.m.). Two‐month‐old wild‐type male mice were used for stereotaxic injection. All animal procedures were performed in accordance with the institutional guidelines of the Animal Care and Use Committee at Central South University.

### Constructs

The 5′‐untranslated region (5′UTR) of transcript variant 1 of *NOTCH2NLC* with 98 GGC repeats was cloned into pX551 vector using the following primers: 5UTR_F: ATA GGT ACC ACC GGT GCT GAG GCG GCG GCC GAG GAG CG and 5UTR_R: ATA GGA TCC CAC AGG GTT CAT AGC CAT CTC GAC ACT GCA ATG CAT GCG CGG GGG TCG CGC A. A 3*HA tag in reading frame with polyglycine (polyG) was then fused to the C‐terminal of 5′UTR to obtain a *NOTCH2NLC*‐(GGC)_98_‐HA construct.

### Stereotaxic injection of AAVs into mouse brain

Stereotaxic surgery was performed as described previously [4]. The location for SN injection was determined according to the distance from bregma: anterior–posterior = −3.1 mm, medial–lateral = ±1.5 mm, dorsal–ventral = −3.9 mm. AAVs of NOTCH2NLC with 98 GGC repeats or green fluorescent protein (GFP) control were injected bilaterally into the SN.

### Antibodies

Primary antibodies used in this study include hemagglutinin (HA; Cell Signaling Technology, 3724S), GFP (Invitrogen, A‐11122), tyrosine hydroxylase (TH; Sigma, AB152), polyG (PEP122) [22], P62 (Abcam, ab56416), P‐α‐synuclein (Abcam, ab184674), and PGP9.5 (Proteintech, 14730‐1‐AP). Secondary antibodies were goat antirabbit, goat antimouse Alexa Fluor 488 or 594 from Jackson ImmunoResearch.

### Cell culture and transfection

Neuro‐2a cells were cultured at 37°C with 5% CO_2_ in Dulbecco modified Eagle medium supplemented with 10% fetal bovine serum and 100 U/mL of penicillin/streptomycin. Plasmid transient transfection on Neuro‐2a cells was performed using Lipofectamine 2000 reagent following the manufacturer's instruction.

### Immunofluorescent staining

Mice were anesthetized and perfused intracardially with 0.9% NaCl, followed by 4% paraformaldehyde. Isolated mouse brains were dehydrated in 30% sucrose and then sectioned at 30 μm. Brain sections were blocked in 3% bovine serum albumin in 0.3% Triton X‐100/phosphate‐buffered saline for 1 h followed by incubation with primary antibodies at 4°C overnight. After incubated with Alexa Fluor‐conjugated secondary antibodies and 4,6‐diamidino‐2‐phenylindole, the brain sections were mounted and visualized with a Zeiss scope.

### Quantification and statistical analysis

All statistical analyses were performed using SPSS 26.0 and R version 4.1.1. All quantification data were presented as mean ± SEM. Fisher exact test was used for genetic analysis. Linear regression and Pearson correlation were used to calculate the relationship between GGC repeat sizes and age at onset of PD, and the correlation coefficient *r* was determined. A *p*‐value of <0.05 was considered statistically significant.

## RESULTS

### Identification of *NOTCH2NLC* GGC repeat expansions in patients with PD

A total of 4327 participants, including 2522 patients diagnosed with PD (1333 men [52.85%], mean [SD] age at onset = 53.54 [11.43] years) and 1085 healthy controls (524 men [48.28%], mean age at study recuiitment = 62.93 [7.15] years) , were recruited. Among the participants, 333 cases had a family history of the disease, of whom 205 had been described and analyzed in our previous publication.

We identified four PD pedigrees (4/333, 1.2%) and three sporadic PD patients (3/2189, 0.14%) carrying pathogenic GGC repeat expansions (≥60 GGC repeats) in the *NOTCH2NLC* gene. The GGC repeat expansion sizes ranged from 66 to 156, with an average of 91.71 ± 27.02 GGC repeats. Notably, we found GGA interruptions at the 3′ end of the GGC repeat expansions were the primary forms of interruptions in these patients with *NOTCH2NLC* GGC expansion.

**FIGURE 1:**
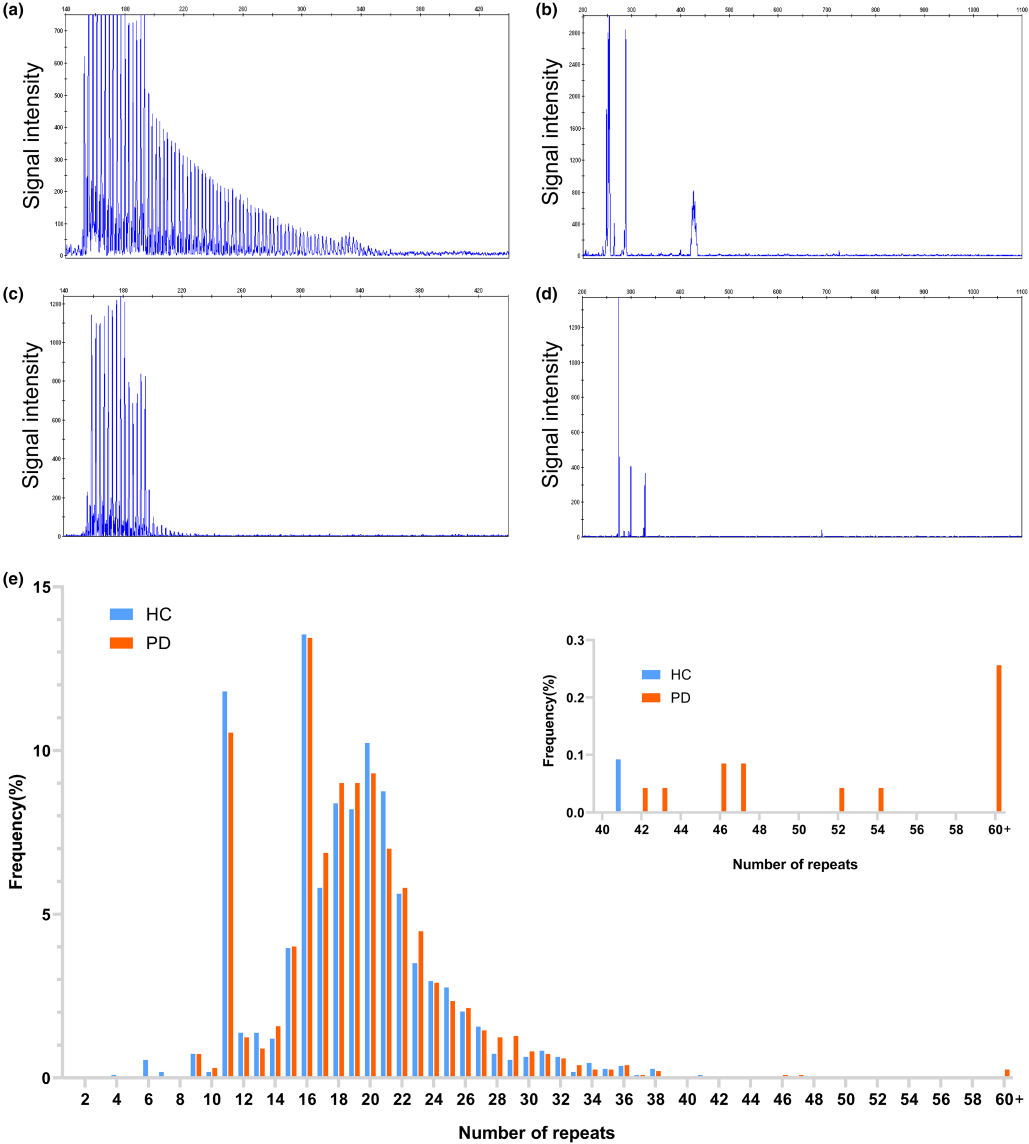
Validation of GGC repeat expansions and repeat sizes in *NOTCH2NLC* gene among patients with Parkinson disease (PD) and healthy control (HC) subjects. Repeat‐primed polymerase chain reaction (RP‐PCR; a) and GC‐rich PCR (GC‐PCR; b) of one patient showed 90 repeat expansions. RP‐PCR (c) and GC‐PCR (d) of one unaffected person showed no repeat expansions. (e) Distribution of *NOTCH2NLC* GGC allele frequency in PD cases and healthy controls. The x‐axis gives the number of GGC repeats, and the y‐axis gives the allele frequency.

Furthermore, eight patients with PD were identified to carry intermediate GGC repeat expansions ranging from 41 to 60 repeats in the *NOTCH2NLC* gene, whereas only one healthy control participant was found to have an intermediate GGC repeat expansions (41 repeats) in this gene. The carrier rate of intermediate GGC repeat expansions in *NOTCH2NLC* among PD patients was slightly higher than that in healthy individuals (0.31% vs. 0.09%, respectively). Despite this, there was no significant difference in the distribution of intermediate *NOTCH2NLC* GGC repeat expansions between PD cases and controls (Fisher exact test *p* = 0.29, odds ratio = 3.459, 95% confidence interval = 0.432–27.690; Figure 1).

### Clinical features of PD patients harboring GGC repeat expansions in *NOTCH2NLC* gene

A total of 13 patients with PD were found to have pathogenic GGC repeat expansions in the *NOTCH2NLC* gene, and eight patients with PD were found to carry intermediate GGC repeat expansions. Those PD patients harboring GGC repeat expansions in *NOTCH2NLC* gene have been confirmed with no pathogenic or likely pathogenic mutations in the established causative genes of PD by whole‐exome sequencing or whole‐genome sequencing and multiplex ligation‐dependent probe amplification in our previous description [5, 6]. The clinical characteristics of these patients are summarized in Table S1.

**FIGURE 2:**
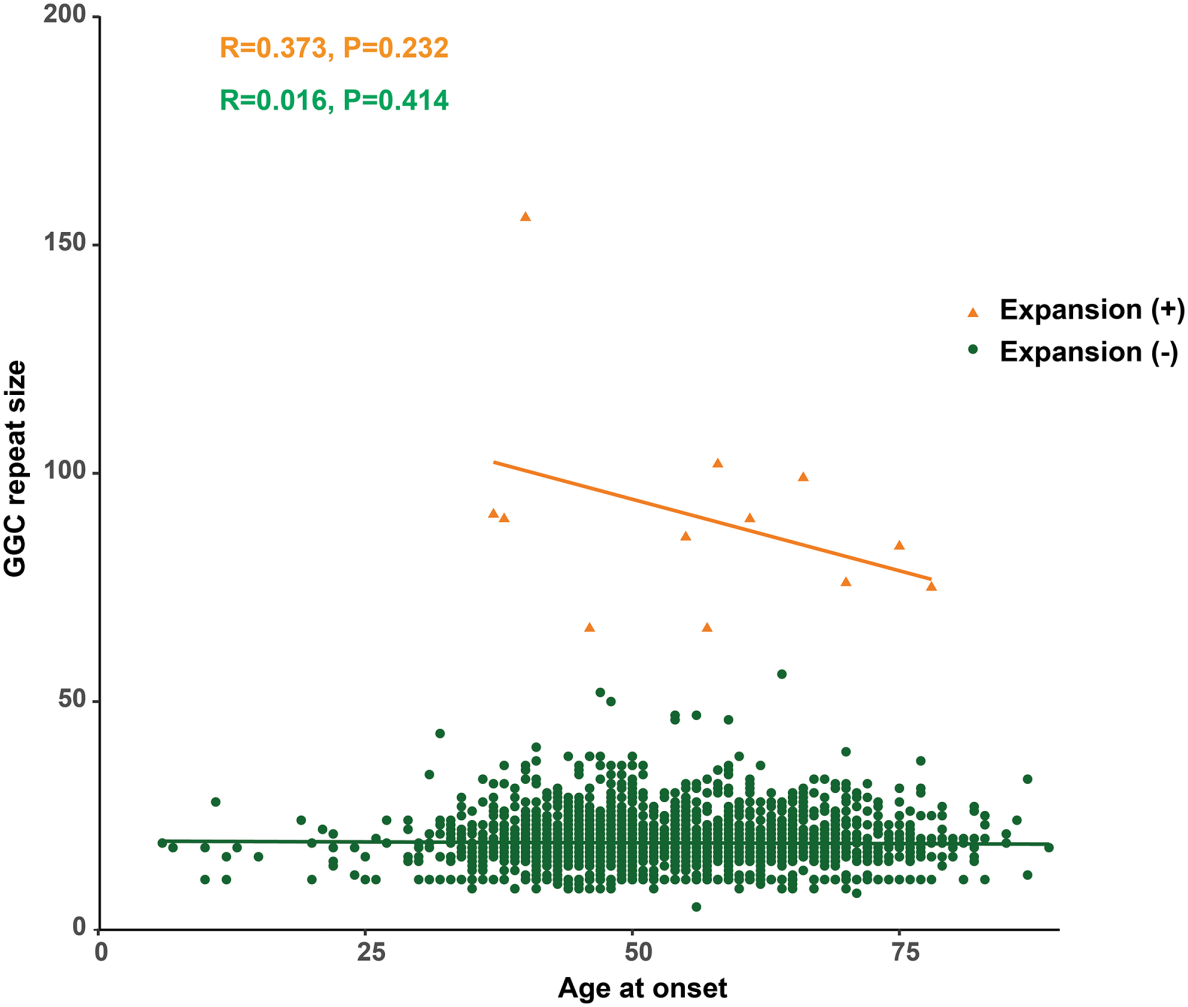
Correlation between age at onset and the size of *NOTCH2NLC* GGC repeat. Linear regression and Pearson correlation were used to calculate the association between GGC repeat sizes and age at onset of PD, and the correlation coefficient *r* was determined. The x‐axis gives the age at onset, and the y‐axis gives the number of GGC repeats. Expansion (+): patients with *NOTCH2NLC* GGC repeat expansions. Expansion (−): patients without *NOTCH2NLC* GGC repeat expansions.

The mean age at onset of these PD patients with pathogenic GGC repeat expansions was 56.75 years (ranging from 38 to 78 years), and the disease duration ranged from 4 to 46 years. The mean age at onset of these PD patients with intermediate GGC repeat expansions was 50.88 years (ranging from 32 to 64 years), and the disease duration ranged from 3 to 16 years. The patients with *NOTCH2NLC* GGC repeat expansions exhibited a wide range of onset ages, and it is currently unclear whether the size of GGC repeat expansions influences the age at onset. Therefore, we evaluated the relationship between GGC repeat size and age at onset in patients with and without *NOTCH2NLC* GGC repeat expansions. No significant correlation between GGC repeat size and age at onset was observed in both expansion carriers (*R* = 0.373, *p* = 0.232) and non‐expansion carriers (*R* = 0.016, *p* = 0.414; Figure 2).

**FIGURE 3:**
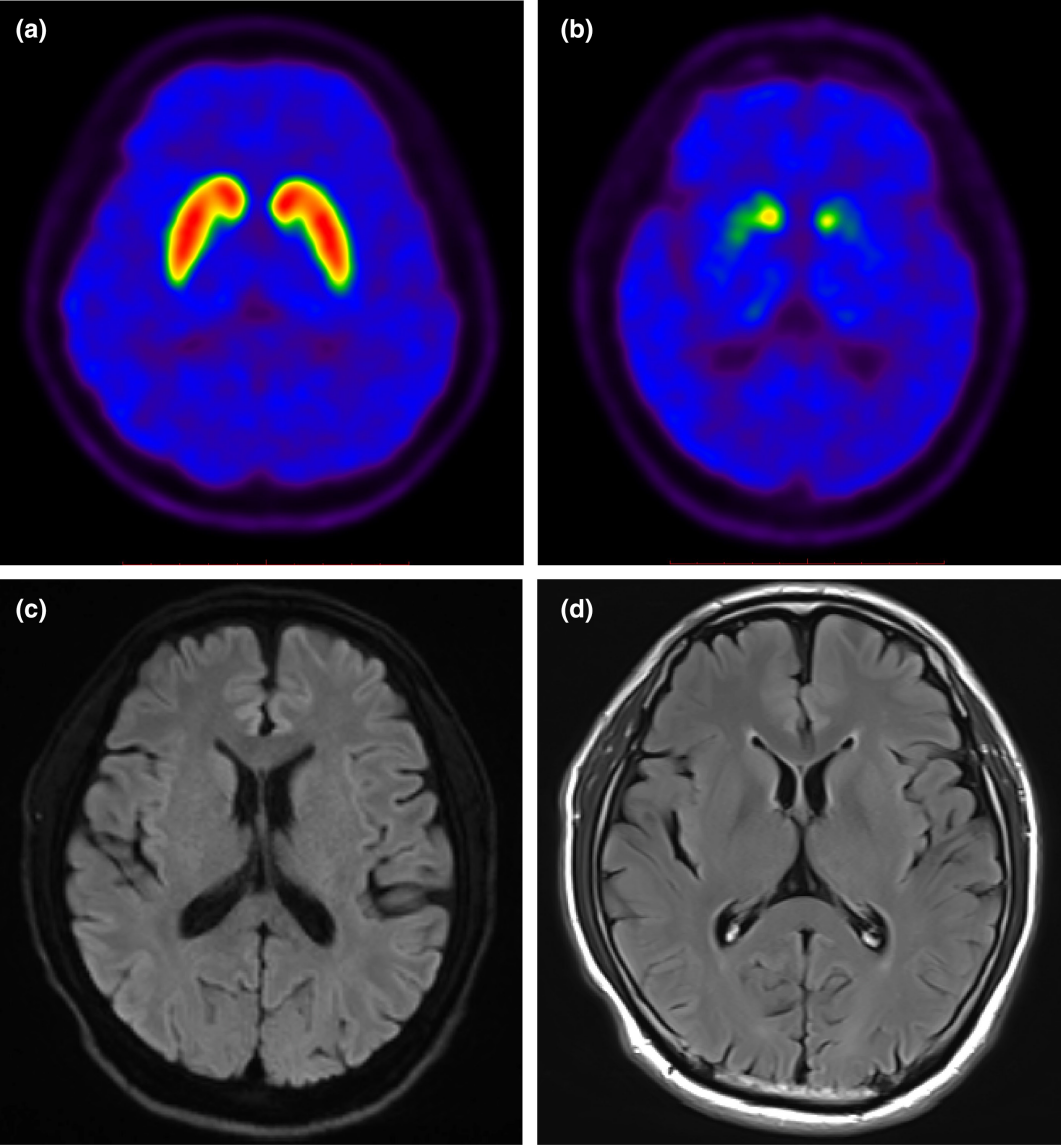
Representative positron emission tomography (PET) and brain magnetic resonance imaging of Parkinson disease harboring *NOTCH2NLC* GGC repeat expansions. Representative axial PET images of ^11^C‐2β‐carbomethoxy‐3β‐(4‐fluorophenyl) tropane (^11^C‐CFT) uptake in a healthy control (a) and PD‐11 (b) show a graded and asymmetrical reduction in dopamine transporter binding (^11^C‐CFT) in the putamen. (c, d) Representative brain diffusion‐weighted imaging images (c) and fluid‐attenuated inversion recovery images (d) of PD‐11, harboring the *NOTCH2NLC* GGC repeat expansions.

All patients with *NOTCH2NLC* GGC repeat expansions were followed up for 3–13 years and were eventually diagnosed with clinically established PD or probable PD according to the Movement Disorder Society clinical diagnostic criteria for PD. Brain magnetic resonance imaging showed white matter hyperintensities in some patients, but cerebellar atrophy was not observed in any patients. ^11^C‐2β‐carbomethoxy‐3β‐(4‐fluorophenyl) tropane positron emission tomography (PET) imaging from case PD‐11 revealed a graded and asymmetrical reduction in dopamine transporter binding in the putamen, which was compatible with PD (Figure 3).

**FIGURE 4:**
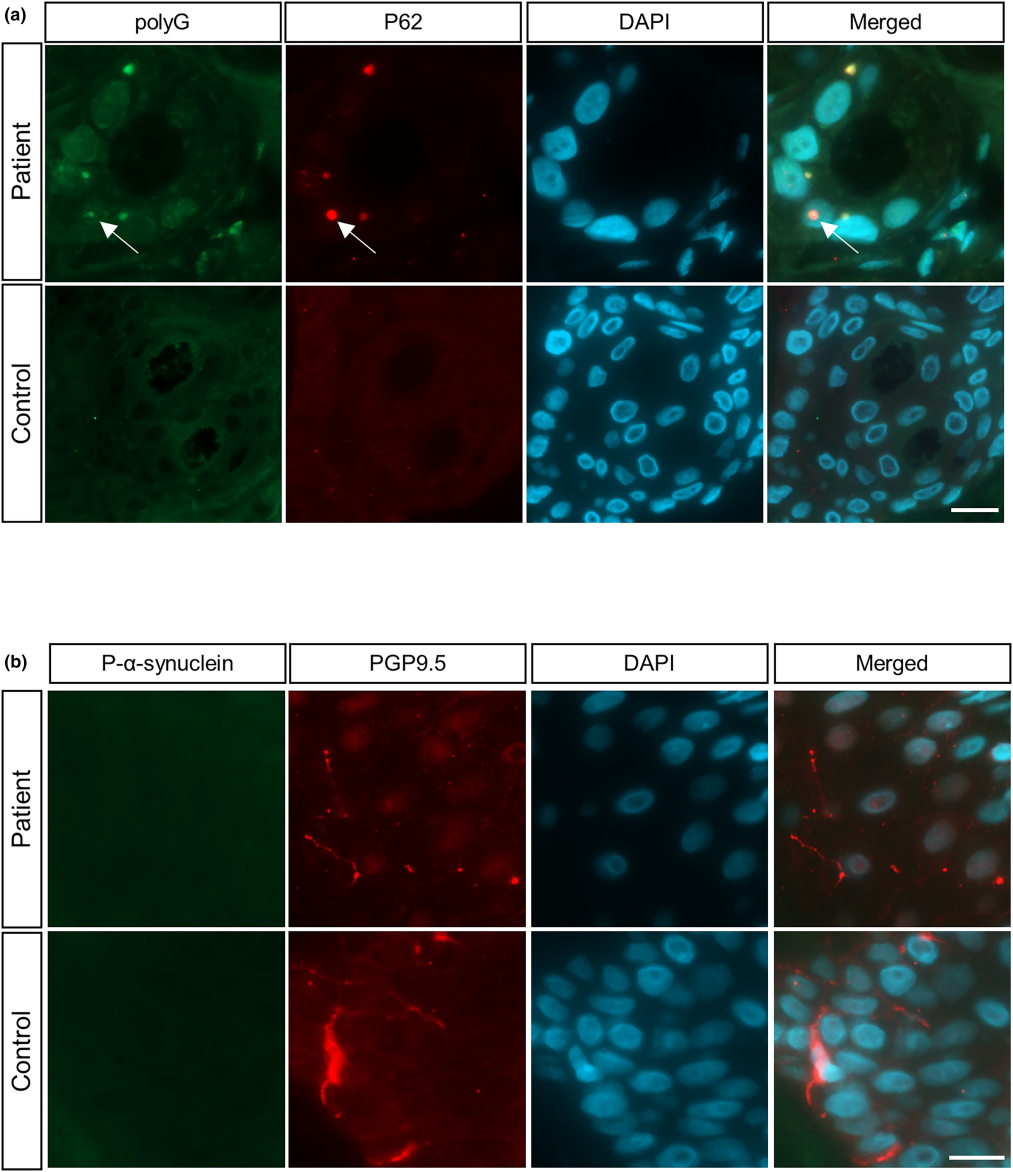
Skin biopsy features of a Parkinson disease (PD) patient harboring GGC repeats in *NOTCH2NLC* and a healthy control. (a) Immunofluorescent staining against polyglycine (polyG) and P62 in the skin of PD‐11 and a healthy control. PolyG intranuclear inclusions colocalized with P62 (white arrows). Green, polyG; red, P62; cyan, 4,6‐diamidino‐2‐phenylindole (DAPI). Scale bar = 10 μm. (b) Immunofluorescent staining against phosphorylated alpha‐synuclein (P‐α‐synuclein) and protein gene product 9.5 (PGP9.5) in the skin of PD‐11 and a healthy control. P‐α‐synuclein colocalized with PGP9.5 in the PD patient with 90 GGC repeats. Green, P‐α‐synuclein; red, PGP9.5; cyan, DAPI. Scale bar = 20 μm.

Furthermore, we examined the histopathological features in the skin biopsy of PD patients. *NOTCH2NLC* with GGC repeat expansion has been reported to produce NOTCH2NLC‐polyG inclusions in NIID patients. However, it remains unknown whether the same histopathological feature exists in PD patients with this mutation. Double‐immunofluorescent staining identified intranuclear polyG inclusions in the skin of case PD‐11 with 90 GGC repeat expansions by using the anti‐PEP122 antibody that was prepared and validated in our previous study [22]. These polyG inclusions were found colocalized with P62, a typical pathological marker in NIID (Figure 4a). Meanwhile, we also detected phosphorylated α‐synuclein in dermal nerve fibers in PD patients but did not find α‐synuclein deposition (Figure 4b).

### Overexpression of *NOTCH2NLC‐(GGC)*_*98*_ causes dopaminergic neuron degeneration in the SN in an age‐dependent manner

**FIGURE 5:**
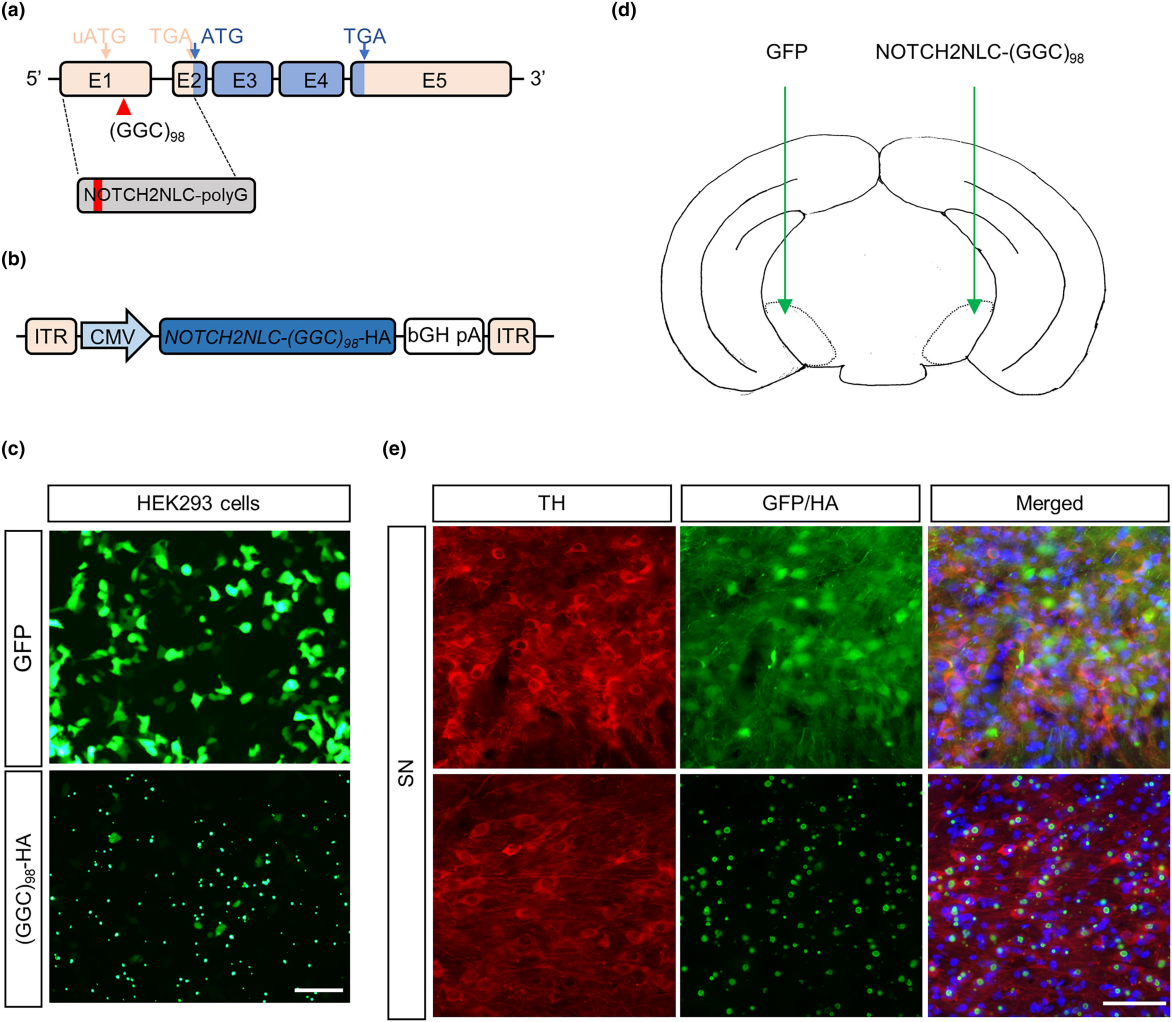
Expression of *NOTCH2NLC‐(GGC)*_*98*_ produces polyglycine inclusions. (a) Schematic diagram of 5′‐untranslated region of *NOTCH2NLC* with 98 GGC repeats fused to hemagglutinin (HA) tag in the glycine frame. (b) Schematic diagram of AAV‐*NOTCH2NLC‐(GGC)*_*98*_ *‐HA* constructs. (c) Immunofluorescence of HEK293 cells transfected with AAV‐*NOTCH2NLC‐(GGC)*_*98*_ *‐HA* and adeno‐associated virus (AAV)–green fluorescent protein (GFP) constructs using anti‐HA antibodies. Green, GFP or HA. Scale bar = 100 μm. (d) Diagram of stereotaxic AAV injection. Indicated AAVs were bilaterally injected into the substantia nigra (SN) of adult wild‐type mice. (e) Immunofluorescent staining against tyrosine hydroxylase (TH) and HA in the brain after injection of AAV‐NOTCH2NLC‐(GGC)_98_‐HA and AAV‐GFP. Green, GFP or HA; red, TH; blue, 4,6‐diamidino‐2‐phenylindole. Scale bar = 50 μm.

Although our genetic findings suggested that GGC repeat expansions in *NOTCH2NLC* were potentially associated with PD, whether these expansions can cause degeneration of dopaminergic neurons in the SN, a key pathological hallmark of PD, remains unknown. To test this possibility, we made a mutant *NOTCH2NLC* construct. An unconventional AUG codon residing in the 5′UTR upstream of the GGC repeats has been verified to produce a short polyG‐containing protein named uN2CpolyG, which has been proved to be the major protein of NOTCH2NLC [22, 23, 24]. We thus cloned the 5′UTR of the *NOTCH2NLC* gene harboring 98 GGC repeats from an NIID patient and fused it with a 3*HA tag to facilitate detection of the polyG‐containing protein (Figure 5a,b).

We first checked the expression of *NOTCH2NLC‐(GGC)*_*98*_ *‐HA* and *GFP* control constructs by transfecting them in cultured HEK293 cells. Immunofluorescent staining showed abundant NOTCH2NLC‐polyG aggregates in cells expressing *NOTCH2NLC‐(GGC)*_*98*_ *‐HA* construct, whereas cells expressing *GFP* control construct exhibited homogeneous cytoplasmic GFP fluorescence (Figure 5c). Then, the *NOTCH2NLC‐(GGC)*_*98*_ *‐HA* and *GFP* control constructs were packaged into AAV9 viruses and bilaterally injected into the SN of adult wide‐type C57BL/6 mice upon stereotaxic injection (Figure 5d). Immunofluorescent staining of HA and GFP verified their expression in the injected brain regions (Figure 5e).

**FIGURE 6:**
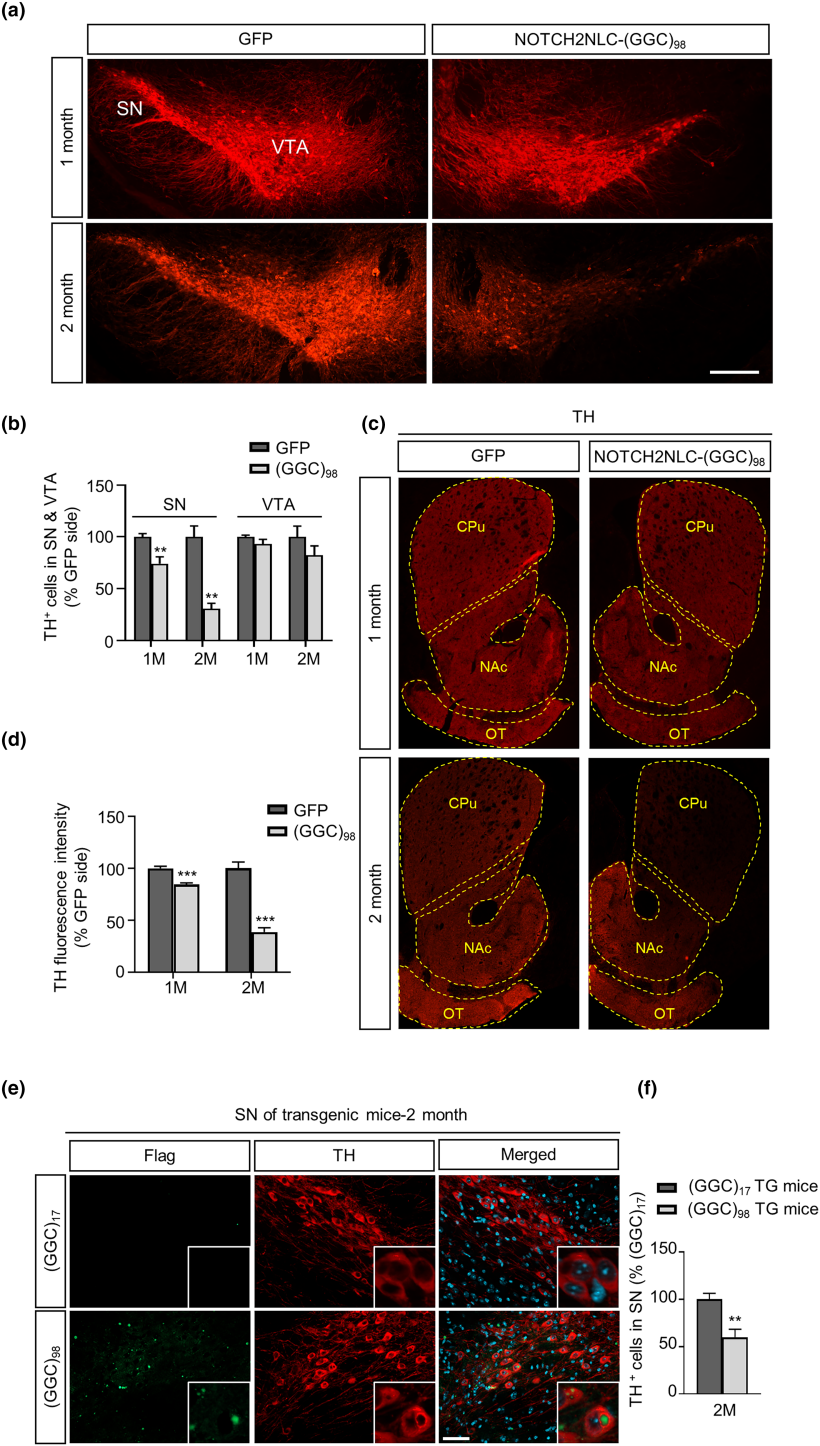
Expression of *NOTCH2NLC‐(GGC)*_*98*_ causes dopaminergic neuron degeneration in the substantia nigra (SN). (a) Immunolabeling of tyrosine hydroxylase‐positive (TH^+^) dopaminergic (DA) neurons in the SN injected with AAV‐NOTCH2NLC‐(GGC)_98_‐HA or adeno‐associated virus (AAV)–green fluorescent protein (GFP). VTA, ventral tegmental area. Scale bar = 100 μm. (b) Quantification of the number of TH^+^ DA neurons in the AAV‐NOTCH2NLC‐(GGC)_98_‐HA‐ or AAV‐GFP‐injected SN and VTA expressed as the percentage change versus the contralateral side. Data are presented as mean ± SEM. **p = 0.0077 (1 month), **p = 0.0042 (2 months), two‐tailed *t*‐test. (c) Immunofluorescent staining against TH in the striatum. CPu, caudate–putamen; NAc, nucleus accumbens; OT, olfactory tubercle. Scale bar = 500 μm. (d) Quantification of the staining intensity of TH in the CPu expressed as the percentage change versus the contralateral side. Data are presented as mean ± SEM. ***p = 0.0008 (1 month), ***p = 0.0002 (2 months), two‐tailed *t*‐test. (e) Immunofluorescent staining against Flag and TH in the SN of 2‐month‐old EIIa‐NOTCH2NLC‐(GGC)_17_ and EIIa‐NOTCH2NLC‐(GGC)_98_ transgenic mice. Green, Flag; red, TH; cyan, 4,6‐diamidino‐2‐phenylindole. SN, substantia nigra. Scale bar = 20 μm. (f) Quantification of the number of TH^+^ DA neurons in the SN of 2‐month‐old EIIa‐NOTCH2NLC‐(GGC)_98_ transgenic mice (TG) expressed as the percentage change versus EIIa‐NOTCH2NLC‐(GGC)_17_ transgenic mice. Data are presented as mean ± SEM. **p = 0.0049, two‐tailed *t*‐test.

Next, we evaluated the effects of GGC repeat expansions within *NOTCH2NLC* on dopaminergic neurons in vivo. One month after injection, expression of NOTCH2NLC‐(GGC)_98_‐HA led to widespread formation of intranuclear or perinuclear polyG inclusions in the dopaminergic neurons labeled with TH. Importantly, TH‐positive (TH^+^) dopaminergic neurons showed reduced fluorescence in the axons and dendrites and had loose distribution in the NOTCH2NLC‐(GGC)_98_‐HA‐injected side compared to the GFP‐injected side (Figure 6a). Additionally, the number of TH^+^ neurons in the SN was significantly reduced by 26% compared to the GFP‐injected side 1 month after injection of NOTCH2NLC‐(GGC)_98_‐HA. The reduction further increased to 69% 2 months after injection (Figure 6b). Interestingly, no significant loss of TH^+^ neurons was found in the ventral tegmental area (Figure 6b).

Because dopaminergic neurons in the SN project processes to the striatum, we then measured the fluorescence intensity of TH immunostaining in the striatum. As expected, the TH signal in the caudate–putamen (CPu) was partially lost, and TH fibers to the nucleus accumbens (NAc) and olfactory tubercle (OT) were indistinguishable from the control side 1 month after NOTCH2NLC‐(GGC)_98_‐HA infection (Figure 6c,d). After 2 months, the TH signal in the CPu was almost completely lost, whereas it was partially preserved in the NAc and OT (Figure 6c,d). Collectively, these findings suggest that the expression of *NOTCH2NLC* with expanded GGC repeats may trigger dopaminergic neuron degeneration in an age‐dependent manner in mice.

Because AAV viral delivery had the potential to induce cellular stress and toxicity to enhance polyG toxicity, we then detected the expression of polyG and the survival of TH^+^ neurons in the SN of EIIa‐NOTCH2NLC‐(GGC)_17_ and EIIa‐NOTCH2NLC‐(GGC)_98_ transgenic mice, which were established in our previous study [22]. As expected, intranuclear or perinuclear polyG inclusions were identified in the TH^+^ neurons of 2‐month‐old EIIa‐NOTCH2NLC‐(GGC)_98_ transgenic mice (Figure 6e). Notably, the number of TH^+^ neurons was reduced significantly in EIIa‐NOTCH2NLC‐(GGC)_98_ transgenic mice when compared to the EIIa‐NOTCH2NLC‐(GGC)_17_ transgenic mice (Figure 6f).

## DISCUSSION

In this study, we conducted a case–control analysis to investigate the association between GGC repeat expansions in the *NOTCH2NLC* gene and PD. Our findings revealed that among a large PD cohort, four PD pedigrees and three sporadic PD patients had pathogenic GGC repeat expansions (>60 repeats) in the *NOTCH2NLC* gene. Our results confirmed that expanded GGC repeats in the *NOTCH2NLC* gene were associated with PD [12, 18]. Furthermore, we performed a longitudinal follow‐up study on 13 carriers with pathogenic *NOTCH2NLC* GGC repeat expansions. Those patients were confirmed with the diagnosis of PD, and they showed typical PD symptoms such as good response to levodopa and dopaminergic neuronal loss detected by dopamine transporter PET. To gain further insights, we will conduct a longitudinal clinicopathological investigation on PD patients carrying these repeat expansions.

In addition, eight PD patients and one healthy control were identified with intermediate‐length *NOTCH2NLC* GGC repeat expansions (41–60 repeats), but the association between the intermediate‐length *NOTCH2NLC* GGC repeat expansions and PD is not well established in our study. It had been reported that intermediate‐length GGC repeat expansions in *NOTCH2NLC* may be associated with PD in two different studies, but such intermediate repeat expansions were very rare in PD cases [18, 19, 20, 27, 28]. The role of intermediate‐length GGC repeat expansions in *NOTCH2NLC* as a genetic risk factor for PD remains uncertain, and further large cohort studies may be required to investigate the significance of *NOTCH2NLC* intermediate‐length GGC repeat expansions in PD.

Our study sheds light on the role of GGC repeat expansions within *NOTCH2NLC* in dopaminergic neurons of the SN in PD. Specifically, we utilized AAV transduction to overexpress *NOTCH2NLC* with expanded GGC repeats and observed widespread intranuclear and perinuclear polyG inclusions, leading to severe dopaminergic neuronal loss in the SN. Importantly, our selective AAV‐NOTCH2NLC‐(GGC)_98_‐HA‐mediated neurodegeneration in the SN is unlikely to be due to nonspecific effects of AAV transduction, because the AAV‐GFP control did not induce neuronal loss in the SN of the same mice. Interestingly, our mouse model showed age‐dependent dopaminergic degeneration, which is consistent with a primary risk factor for neurodegenerative diseases such as Alzheimer disease, PD, and other repeat‐expansion‐mediated diseases. Given that neurons are terminally differentiated cells that do not divide, aging‐related factors, including greater vulnerability and reduced protein homeostasis, make the neurons more susceptible to damage than glia cells. According to current reports, the potential mechanism of NOTCH2NLC‐polyG‐mediated toxicity in our mouse model may be due to DNA repair alterations or nucleocytoplasmic transport disruption, which was explored in the cell and mouse models of NIID, another neurodegenerative disorder caused by the same mutation in *NOTCH2NLC* [22, 23, 24, 25]. In addition, like other misfolded proteins, accumulated NOTCH2NLC‐polyG protein aggregates may recruit, through protein–protein interactions, other proteins such as RNA‐binding proteins and transcription factors, thereby sequestering them from their functional locations and affecting their activities. Furthermore, widespread of NOTCH2NLC‐polyG inclusions may cause neuronal death from proteotoxic stress, abnormalities in mitochondrial dysfunction, ubiquitin–proteasomal and autophagosomal–lysosomal systems, and neuroinflammation [17, 22, 23, 24, 25, 29]. Future studies are necessary to further investigate the molecular pathogenesis of PD.

We also examined the phosphorylated alpha‐synuclein in SN but did not find positive signals in the mice injected with AAV‐NOTCH2NLC‐(GGC)_98_. Our data may indicate that polyG inclusions did not accelerate phosphorylated alpha‐synuclein deposition at least 2 months after injection of AAV‐NOTCH2NLC‐(GGC)_98_. However, GGC repeat expansion of NOTCH2NLC may impact alpha‐synuclein accumulation or clearance over time. It is well known that repeat expansion disorders such as Huntington disease and spinocerebellar ataxia types 1–3 have been found to be associated with dysfunction of the autophagy system, and they often involve abnormal folding and aggregation of proteins, resulting in disturbance of protein homeostasis within the cell. Thus, it is possible that the clearance of other proteins such as alpha‐synuclein will be affected, and the phenotype may become more significant over time. However, further research is required to explore the impact of GGC repeat expansions in *NOTCH2NLC* and alpha‐synuclein.

The GGC repeat expansions of *NOTCH2NLC* is found in many diseases, including neuronal intranuclear inclusion disease, essential tremor, PD, Alzheimer disease, multiple system atrophy, and even oculopharyngodistal myopathy. How could the GGC repeat expansion of *NOTCH2NLC* result in different disease phenotypes? It seems that GGC repeat size and the types of trinucleotide interruption may lead to different phenotypes of disease. Generally, the repeat size of muscle weakness‐dominant phenotype is largest, and parkinsonism‐dominant phenotype is smallest. Dementia‐dominant and essential tremor‐dominant phenotypes usually have a purer GGC repeat. There are also other possible mechanisms, including the toxicity of polyG‐containing protein, the toxicity of repeat RNA, and the methylation status of *NOTCH2NLC* [17, 22].

In conclusion, our study provides genetic and functional evidence to support the role of GGC repeat expansions in *NOTCH2NLC* in the pathogenesis of PD.

## AUTHOR CONTRIBUTIONS

**Zhenhua Liu:** Conceptualization; investigation; methodology; data curation; supervision; formal analysis; visualization; funding acquisition; writing – original draft; writing – review and editing; project administration. **Qiong Liu:** Writing – original draft; data curation; methodology; conceptualization; formal analysis; visualization; writing – review and editing; software; validation. **Juan Chen:** Methodology; data curation. **Jin Xue:** Methodology; data curation; formal analysis; investigation; software; validation. **Xun Zhou:** Methodology; formal analysis; data curation; investigation; visualization. **Yun Tian:** Methodology; data curation. **Qiao Xiao:** Data curation; methodology. **Wen Huang:** Data curation; methodology. **Yongcheng Pan:** Methodology; data curation. **Xiaoxia Zhou:** Data curation; formal analysis. **Jian Li:** Methodology; data curation. **Yuwen Zhao:** Methodology; data curation; formal analysis; software. **Hongxu Pan:** Data curation. **Yige Wang:** Data curation. **Runcheng He:** Data curation. **Yaqin Xiang:** Data curation. **Tian Tu:** Data curation. **Qian Xu:** Methodology; data curation; resources. **Qiying Sun:** Methodology; data curation; resources. **Jieqiong Tan:** Data curation. **Xinxiang Yan:** Methodology; data curation; resources. **Jinchen Li:** Data curation. **Jifeng Guo:** Methodology; data curation; resources; writing – review and editing. **Lu Shen:** Methodology; data curation; resources; writing – review and editing. **Ranhui Duan:** Methodology; formal analysis; investigation; visualization; data curation; validation; supervision; writing – review and editing. **Beisha Tang:** Conceptualization; supervision; resources; writing – review and editing; funding acquisition.

## FUNDING INFORMATION

This study was supported by the National Natural Science Foundation of China (grants 82371274, 82001359, U20A20355, 32071037), the National Key Research and Development Program of China (grants 2016YFC306000, 2021YFC2501204), the Scientific Research Project of Hunan Provincial Health Commission (grant 202203074637), Hunan Innovative Province Construction Project (grants 2019SK2335, 2021SK1010), and Hunan Provincial Natural Science Foundation of China (grants 2022JJ40803, 2021JJ41009, 2023JJ10097, 2021JJ41014).

## CONFLICT OF INTEREST STATEMENT

None of the authors has any conflict of interest to disclose.

## ETHICS STATEMENT

The study was endorsed by the ethics committee of Xiangya Hospital of Central South University. All participants gave informed consent prior to inclusion in PD‐MDCNC.

## Supporting information


TABLE S1

## Data Availability

The data that support the findings of this study are available from the corresponding author upon reasonable request.
