# Supplementary material for: GGC expansions in NOTCH2NLC contribute to Parkinson disease and dopaminergic neuron degeneration
Source: Eur J Neurol. 2023 Nov 17;31(2):e16145. doi: 10.1111/ene.16145 (PMC11235938; doi:10.1111/ene.16145)
Supplement: Supplementary file 1 — TABLE S1 [file ENE-31-e16145-s001.docx]

Table S1. Characteristics of patients with Parkinson’s disease harboring *NOTCH2NLC* CGG expansions

| Sample | Sex | Age at onset, years | Disease duration, years | Repeat sizes |
| --- | --- | --- | --- | --- |
| PD-1 | Male | 75 | 5 | 84 |
| PD-2 | Male | 58 | 15 | 102 |
| PD-3 | Male | 66 | 5 | 99 |
| PD-4 | Male | 78 | 4 | 75 |
| PD-5 | Male | 37 | 18 | 91 |
| PD-6 | Male | 46 | 8 | 66 |
| PD-7 | Male | 58 | 10 | 70 |
| PD-8 | Female | 66 | 11 | 76 |
| PD-9 | Male | 61 | 7 | 200 |
| PD-10 | Female | 40 | 46 | 156 |
| PD-11 | Male | 38 | 12 | 90 |
| PD-12 | Male | 55 | 14 | 86 |
| PD-13 | Male | 56 | 6 | 66 |
| PD-14 | Female | 64 | 9 | 56 |
| PD-15 | Male | 47 | 17 | 52 |
| PD-16 | Male | 48 | 11 | 50 |
| PD-17 | Male | 56 | 16 | 47 |
| PD-18 | Male | 50 | 9 | 46 |
| PD-19 | Female | 56 | 12 | 46 |
| PD-20 | Male | 32 | 3 | 43 |
| PD-21 | Female | 54 | 4 | 47 |
